# Tuina for Enuresis in Children: A Systematic Review and Meta-Analysis of Randomized Controlled Trials

**DOI:** 10.3389/fpubh.2022.821781

**Published:** 2022-04-12

**Authors:** Chiin Tong, Qida He, Manin Ho, Zhenghong Zhong, Qibiao Wu, Min Chen

**Affiliations:** ^1^Faculty of Chinese Medicine, Macau University of Science and Technology, Taipa, Macau SAR, China; ^2^State Key Laboratory of Quality Research in Chinese Medicines, Macau University of Science and Technology, Taipa, Macau SAR, China; ^3^Zhuhai MUST Science and Technology Research Institute, Zhuhai, China; ^4^Guangdong-Hong Kong-Macao Joint Laboratory for Contaminants Exposure and Health, Guangzhou, China

**Keywords:** Tuina, massage, enuresis in children, randomized controlled trial, meta-analysis

## Abstract

**Objective:**

To evaluate the effects of Tuina (massage) vs. non-Tuina traditional Chinese medicine (TCM) treatments on nocturnal enuresis in children.

**Methods:**

A systematic review and meta-analysis of randomized controlled trials (RCTs) was conducted following the Preferred Reported Items for Systematic Review and Meta-analysis (PRISMA) guidelines. RevMan 5.3 software was used for meta-analysis.

**Results:**

Twelve RCTs recruiting a total of 1,007 children were included. Meta-analysis results showed that, compared with non-Tuina TCM treatments, Tuina could significantly improve the total effective rate of children's enuresis [RR = 1.29, 95%CI (1.22–1.36), *P* < 0.00001]. The results of subgroup analyses indicated that the total effective rate of Tuina combined with acupuncture in the treatment of nocturnal enuresis was higher than acupuncture alone [RR = 1.24, 95%CI (1.12–1.37), *P* < 0.0001]. The total effective rate of Tuina in the treatment of enuresis in children was better than that of herbal medicine alone [RR = 1.45, 95%CI (1.31–1.61), *P* < 0.00001]. The total effective rate of Tuina combined with herbal medicine in the treatment of enuresis in children was better than that of herbal medicine alone [RR = 1.16, 95%CI (1.06–1.26), *P* = 0.0007]. No adverse reactions of Tuina were reported in all included studies.

**Conclusion:**

From the available evidence, Tuina, or Tuina combined with non-Tuina TCM treatments (acupuncture, or herbal medicine) can improve the clinical outcome of children with enuresis, indicating Tuina is a promising treatment choice for children's enuresis. However, because of the intrinsic limitations of the included studies, more high-quality randomized controlled trials with longer follow-up are still needed to further confirm the efficacy and safety of Tuina in the treatment of nocturnal enuresis in children.

## Introduction

Enuresis, also known as “bed-wetting”, is defined as intermittent urinary incontinence during sleep in children older than age 3, which is also the definition recognized and adopted by traditional Chinese medicine (TCM) (1, 2). Primary enuresis in children is a common childhood disease with heterogeneous and complex etiology. The delay of central nervous system function maturation, genetic factors, sleep disorders, and abnormal secretion of the antidiuretic hormone can lead to the occurrence of primary enuresis in children (3). Children with long-term enuresis may have impaired immune function, psychological disorders, low self-esteem, and other problems, which also lead to negative effects on the quality of life of their parents (4).

Clinically, anticholinergic drugs, tricyclic antidepressants, artificial antidiuretics, and central stimulants are often used for the treatment of enuresis, but their efficacy is poor and often leads to many side effects (5). Desmopressin, for example, has the most important side effects of water poisoning and hyponatremia, and a high relapse rate once discontinued (6). TCM treatments, such as herbal medicine, acupuncture and moxibustion, and Tuina are commonly used for the treatment of nocturnal enuresis, and many clinical studies have shown that they are effective and have great potential in the treatment of this disorder (7–11). However, due to the unpleasant taste or invasiveness, non-Tuina TCM treatments including herbal medicine, acupuncture and moxibustion have unsatisfactory compliance in young children (12, 13), on the contrary, Tuina is non-invasive, safe, and comfortable, making it more suitable and acceptable for children with nocturnal enuresis (14). Tuina for children originated from adult Tuina, but because children's body is immature, their skin and flesh are tender, the force of Tuina for children is much gentler than that for adults (15). However, whether Tuina is more or less effective than non-Tuina TCM treatments for the treatment of children's enuresis remains unknown, therefore, this systematic review and meta-analysis included the related randomized controlled trials (RCT) to investigate the difference in the effects of Tuina vs. non-Tuina TCM treatments for enuresis in children, aiming to provide evidence for clinicaldecision-making.

## Materials and Methods

This systematic review and meta-analysis were performed following the PRISMA (Preferred Reported Items for Systematic Review and Meta-analysis) guidelines (16).

### Source

The electronic databases including China national knowledge infrastructure (CNKI), Wanfang Database, Chongqing VIP, The Cochrane Library, and PubMed were searched for potentially eligible studies.

### Retrieval Strategy

A combination of the following English terms was used to search the English databases: (“enuresis” OR “bed-wetting”) AND (“pediatric” OR “pediatric” OR “adolescent” AND “child” OR “children” OR “kid” OR “youngster”) AND (“Tuina” OR “massage”). For the Chinese databases, the following keywords were used in combined ways: [“yiniao (enuresis)” OR “Niaochuang (bed-wetting)” AND (“Xiaoer (children)” OR “ertong (children)” OR “qingshaonian (adolescent)”) AND (“Tuina” OR “Anmo (massage)”]. The retrieval time was from the establishment to July 2021.

Furthermore, the reference lists of all the related articles were reviewed to identify potential RCTs. There were no trials excluded due to their publication status or language.

### Inclusion Criteria

(1) Participants: All the participants should meet the following diagnostic criteria of pediatric enuresis (17): The onset age is 3–18 years old, bed-wetting every night or every few days, or even countless bed-wetting overnight (1, 2, 18). (2) Interventions: The experimental group was treated with Tuina, or Tuina combined with non-Tuina TCM treatment (herbal medicine, acupuncture, etc.); the control group was treated with non-Tuina TCM treatment alone. Except for the Tuina treatment, other treatments were the same in the control group and the experimental group. (3) Type of studies: Randomized controlled trials, whether or not allocation concealment, blinding methods were used. (4) Outcome measures: There were clear and well-recognized criteria for efficacy evaluation, the total effective rate (TER) was regarded as the primary outcome, and adverse reactions were the secondary outcome.

### Exclusion Criteria

(1) Non-randomized controlled trials (non-RCTs); (2) Tuina was not the intervention; (3) Literature of missing outcome indicators; (4) Duplicated studies, reviews, studies with incomplete data and unable to obtain the full text; (5) Enuresis caused by urinary tract infection, or severe liver and kidney dysfunction.

### Outcome Measures

#### Total Effective Rate

According to the standards for diagnosis and efficacy of diseases and syndromes of traditional chinese medicine (17), the treatment efficacy was evaluated and graded after treatment:

a) Complete remission (CR): The patient's enuresis disappeared and no recurrence within one month;
b) Significantly effective (SE): Enuresis symptoms were significantly relieved, and recurrence occurred within a month, but less than 1 time per week;
c) Partial remission (PR): Enuresis frequency decreased, more than once a week;
d) Ineffective: No change in enuresis frequency compared with that before treatment.

The total effective rate was defined as the percentage of patients who achieved CR or SE or PR.

#### Adverse Reactions

Retinal hemorrhage, fracture, hematoma, ecthyma, vomiting, etc., related to the treatments (19).

### Study Selection

All the candidate articles were screened by two independent investigators (C.I. TONG and M.I. HO) based on the title and abstract. The full texts were retrieved for further evaluation according to the inclusion and exclusion criteria. All inclusion disagreements were resolved by consensus.

### Quality Assessment of Included Studies

The included studies were assessed for risk bias using the Review Manager5.3 software built-in risk bias assessment tool provided by the Cochrane Handbook V.5.1.0 (Cochrane Collaboration) (20). The evaluation items included the generation of random sequences, allocation concealment, blinding of investigators and subjects, blind evaluation of study outcomes, the integrity of outcome data, selective reporting of study results, and other sources of risk bias. The bias risk was assessed as low risk, unknown risk, and high risk according to the quality of the included studies. The characteristics of all included RCTs are summarized in Table 1.

**Table 1:** Principal characteristics of the studies included in the meta-analysis.

**Reference**	**Design**	**Jadadscore**	**Sample size (T/C)**	**Outcome measures**	**Interventions**		**Course of treatment(day)**
					**Treatment group**	**Control group**	
Chen (21)	RCT	2	40/40	TER	TA	AC	6
Deng (22)	RCT	4	41/40	TER, TCMJQS	TH	HM	7
Fan (23)	RCT	2	32/28	TER	TA	AC	10
Hu (24)	RCT	3	30/30	TER, TCMJQS	TA	AC	6
Jiang (25)	RCT	3	48/47	TER	TN	HM	30
Lai (26)	RCT	3	40/40	TER	TH	HM	15
Liu (27)	RCT	2	100/100	TER	TN	HM	7
Wang (28)	RCT	2	28/28	TER	TN	HM	6
Xi, (29)	RCT	4	55/55	TER, TCMJQS	TH	HM	28
Xie, (30)	RCT	3	30/30	TER, TCMJQS	TH	HM	5
Yan, (31)	RCT	3	30/30	TER, TCMJQS	TN	HM	10
Liu (27)	RCT	2	35/30	TER	TA	AC	7

*RCT, randomized controlled trial; T/C, treatment group/control group; TN, Tuina; AC, Acupuncture; HM, herbal medicine; TA, Tuina plus Acupuncture; TH, Tuina plus herbal medicine; TER, Total effect rate; TCMJQS, Traditional Chinese medicine grading quantitative scoring*.

### Statistical Analysis

The included studies were analyzed using Review Manager 5.3 software provided by the Cochrane Collaboration. The *I*^2^ statistic and Chi^2^ test were used to determine statistical heterogeneity. If *P* > 0.1 and *I*^2^ <50%, the heterogeneity was considered acceptable and the fixed-effect model was adopted. If *P* ≤ 0.1 and *I*^2^≥ 50%, substantial heterogeneity was considered, and the random-effects model was used to calculate the pooled effect size, and the source of heterogeneity would be analyzed. According to data types, the risk ratio (RR) or odds ratio (OR) were used for dichotomous data, and the weighted mean difference (WMD) or standardized mean difference (SMD) for continuous data. In total 95% confidence interval (CI) was calculated for both (32–34).

### Risk of Bias Across Trials

The funnel plots and Egger's test were carried out to assess the potential bias when there were more than 10 trials included in a meta-analysis (20, 35, 36).

## Results

Table 2 summarizes the results of the meta-analysis.

**Table 2:** Summary of the meta-analysis (pooled data across categories in the control group).

**Outcome or subgroup**		**No. of studies**	**Participants**	**Statistical method**	**Effect size**	***P***
**Non-Tuina TCM treatments**		12	1007	RR (fixed), 95% CI OR (fixed), 95% CI RD (fixed), 95% CI	1.29 [1.122, 1.36] 6.61[4.25, 10.27] 0.21 [0.17, 0.25]	<0.00001* <0.00001* <0.00001*
**TA vs. AC**		4	265	RR (fixed), 95% CI	1.24 [1.12, 1.37]	<0.0001*
				OR (fixed), 95% CI	6.61[2.61, 16.76]	<0.0001*
				RD (fixed), 95% CI	0.18 [0.10, 0.26]	<0.00001*
**TN vs. HM**		4	411	RR (fixed), 95% CI	1.45 [1.31, 1.61]	<0.00001*
				OR (fixed), 95% CI	11.60[5.58,24.14]	<0.00001*
				RD (fixed), 95% CI	0.30 [0.23, 0.37]	<0.00001*
**TH vs. HM**		4	331	RR (fixed), 95% CI	1.16 [1.06, 1.26]	0.0007*
				OR (fixed), 95% CI	3.37 [1.64, 6.92]	0.001*
				RD (fixed), 95% CI	0.13 [0.06, 0.20]	0.0004*
**Sensitive** **analyses**	**High-quality study** (Jadad score ≥ 3)	7	546	RR (fixed), 95% CI	1.26 [1.17, 1.36]	<0.0001*
				OR (fixed), 95% CI	5.12 [2.93, 8.94]	<0.0001*
				RD (fixed), 95% CI	0.19 [0.13, 0.25]	<0.0001*
	**Period of treatment** (≥15 days)	6	465	RR (fixed), 95% CI	1.31[1.20, 1.43]	<0.0001*
				OR (fixed), 95% CI	5.50 [3.08, 9.82]	<0.0001*
				RD (fixed), 95% CI	0.22 [0.15, 0.28]	<0.0001*
	**Date of publication** (In the recent 6 years)	7	542	RR (fixed), 95% CI	1.24 [1.15, 1.33]	<0.00001*
				OR (fixed), 95% CI	5.92 [3.17, 11.05]	<0.00001*
				RD (fixed), 95% CI	0.18 [0.13, 0.24]	<0.00001*
	**Sample size** (≥ 40 /group)	6	646	RR (fixed), 95% CI	1.28 [1.20, 1.37]	<0.00001*
				OR (fixed), 95% CI	6.57 [3.76, 11.49]	<0.00001*
				RD (fixed), 95% CI	0.21 [0.16, 0.26]	<0.00001*

*TN, Tuina; AC, Acupuncture; HM, herbal medicine; TA, Tuina plus Acupuncture; TH, Tuina plus herbal medicine*.

### Study Selection

**Figure 1:**
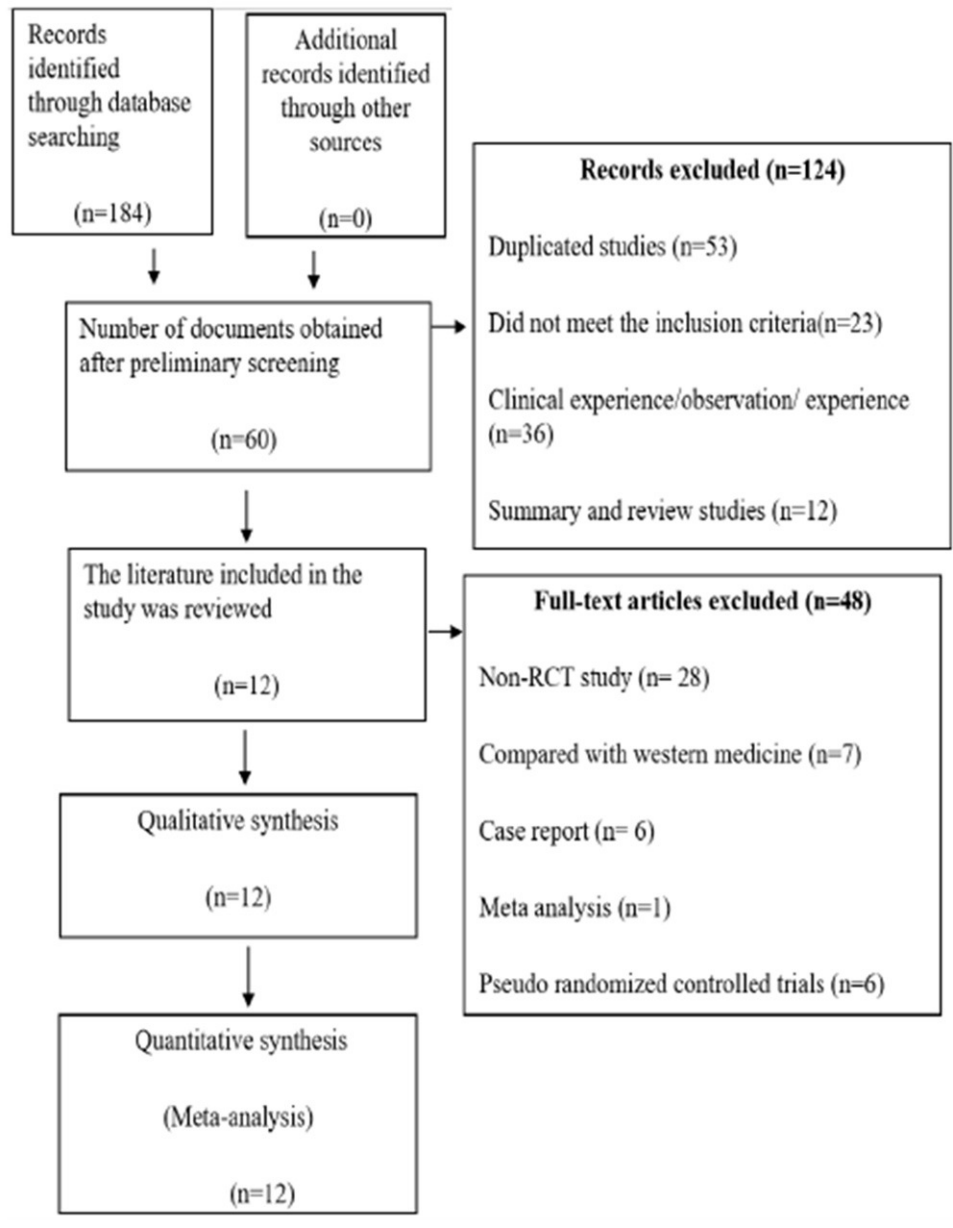
Preferred reporting items for systematic reviews and meta-analysis (PRISMA) search diagram.

A total of 184 articles in Chinese and English were retrieved. In total 124 duplicates studies, Summary and review studies, did not meet the inclusion criteria and empirical articles. According to the inclusion and exclusion criteria, 12 references were finally obtained. After reading the full texts, the final number of studies included in quantitative analysis was 12 (21–31, 37). The literature screening process is shown in Figure 1. The characteristics of the tests are shown in Table 1.

### Methodologic Quality

**Figure 2:**
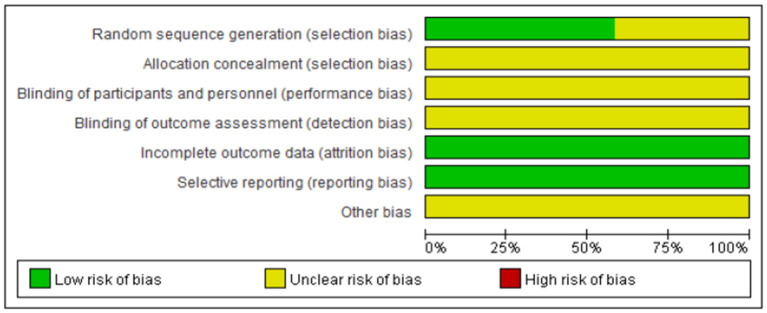
Risk of bias graph.

**Figure 3:**
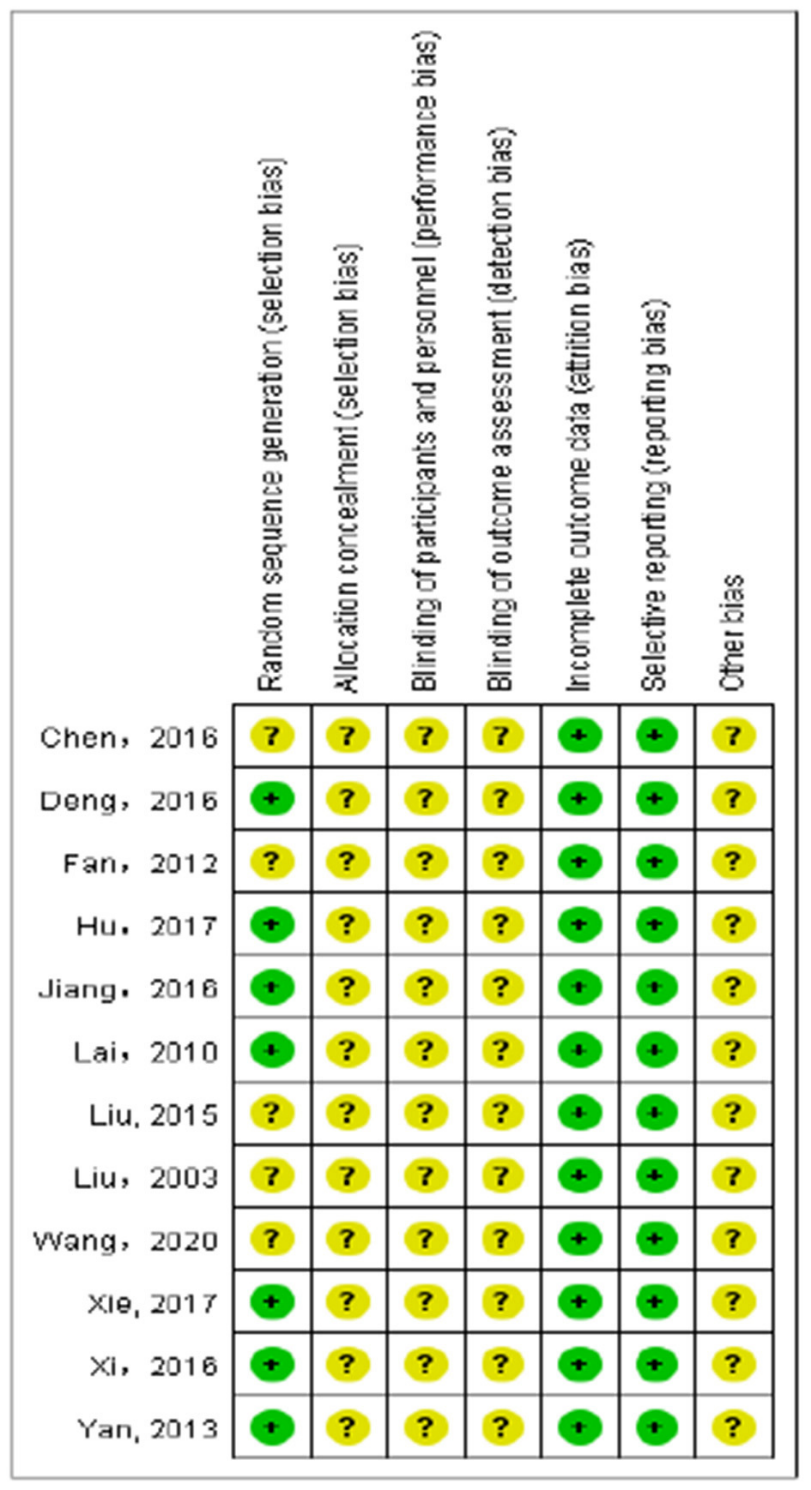
Risk of bias summary.

Randomized method was used in all the 12 included studies (21–31, 37), but only 7 (21–26, 28–31) clearly described the randomized methods (table of random numbers). No studies clearly described the allocation concealment, the blind method of investigator, participants, and outcome assessment. There was no case of patient withdrawal or loss of follow-up in all the studies. The overall quality of RCTs was not high. The risk of bias and methodological quality of the included RCTs was assessed and shown in Tables 1, 3 and Figures 2, 3.

**Table 3:** The methodologic quality of the included trials was assessed using the Cochrane risk of bias tool.

**Reference**	**Random sequence generation**	**Allocation concealment**	**Blinding of participants and personnel**	**Blinding of outcome assessment**	**Incomplete outcome data**	**Selective reporting**	**Other bias**
Chen (21)	?	?	?	?	+	+	?
Deng (22)	+	?	?	?	+	+	?
Fan (23)	?	?	?	?	+	+	?
Hu (24)	+	?	?	?	+	+	?
Jiang (25)	+	?	?	?	+	+	?
Lai (26)	+	?	?	?	+	+	?
Liu (27)	?	?	?	?	+	+	?
Wang (28)	?	?	?	?	+	+	?
Xi, (29)	+	?	?	?	+	+	?
Xie, (30)	+	?	?	?	+	+	?
Yan, (31)	+	?	?	?	+	+	?
Liu (27)	?	?	?	?	+	+	?

*+ = low risk of bias; ? = unclear risk of bias; - = high risk of bias*.

### Clinical Efficacy

#### Total Effective Rate

**Figure 4:**
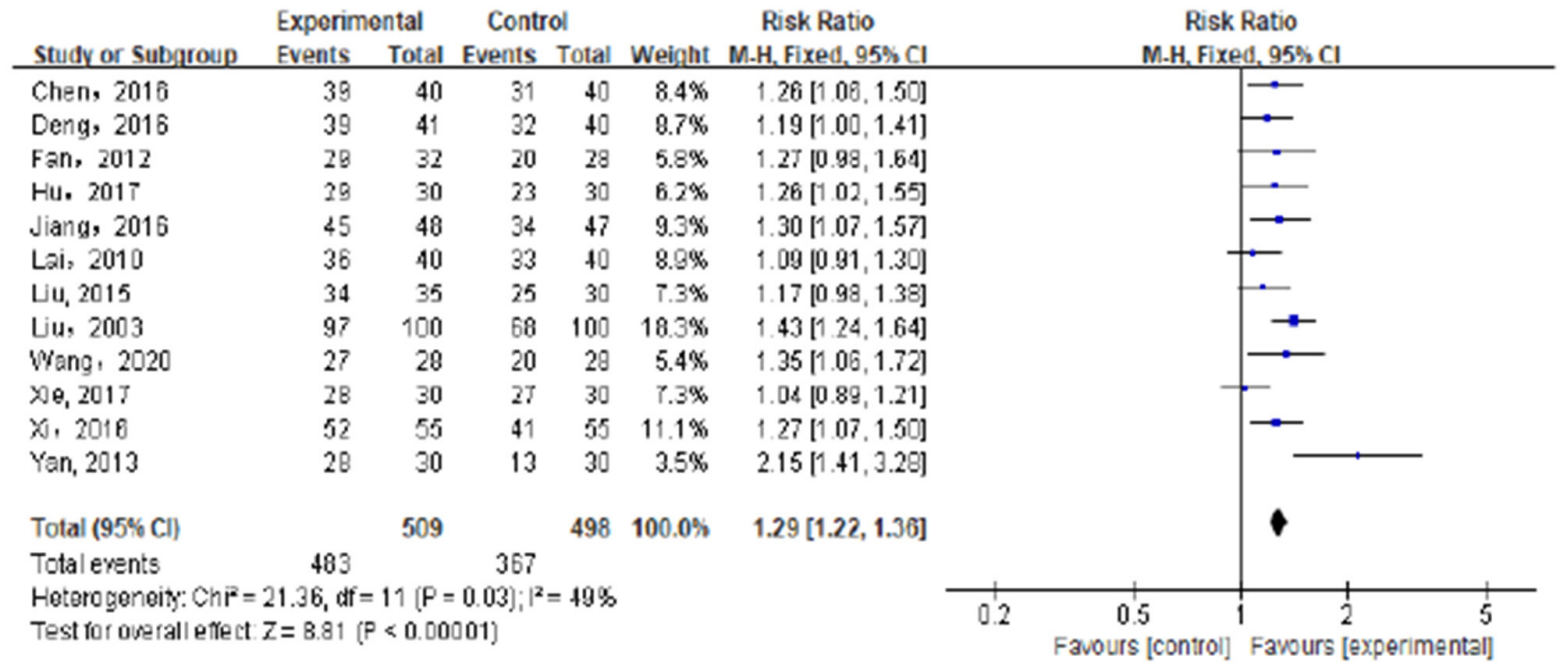
Compared with the control group, the total effective rate of the experimental group was significantly improved.

There were 12 trials involving 1,007 patients (21–31, 37) that compared the total effective rate of Tuina vs. non-Tuina TCM treatments in the treatment of pediatric enuresis. There was no significant heterogeneity among the included studies (*I*^2^ = 49%), the fixed effects model was used for meta-analysis. The results showed that the total effective rate of the Tuina treatment group was higher than that of the non-Tuina control group [RR = 1.29, 95%CI (1.22–1.36), *P* < 0.00001], and the difference between the two groups was statistically significant. As shown in Figure 4.

#### Subgroup Analysis

The subgroup analyses were conducted to determine the causes of heterogeneity among the results of studies. The subgroup analysis based on the method of TCM treatments showed that different method of TCM treatments was the cause of heterogeneity and the clinical efficacy of Tuina was better than other non-Tuina TCM treatments in the management of children's enuresis.

##### Tuina Plus Acupucture vs. Acupuncture

**Figure 5:**
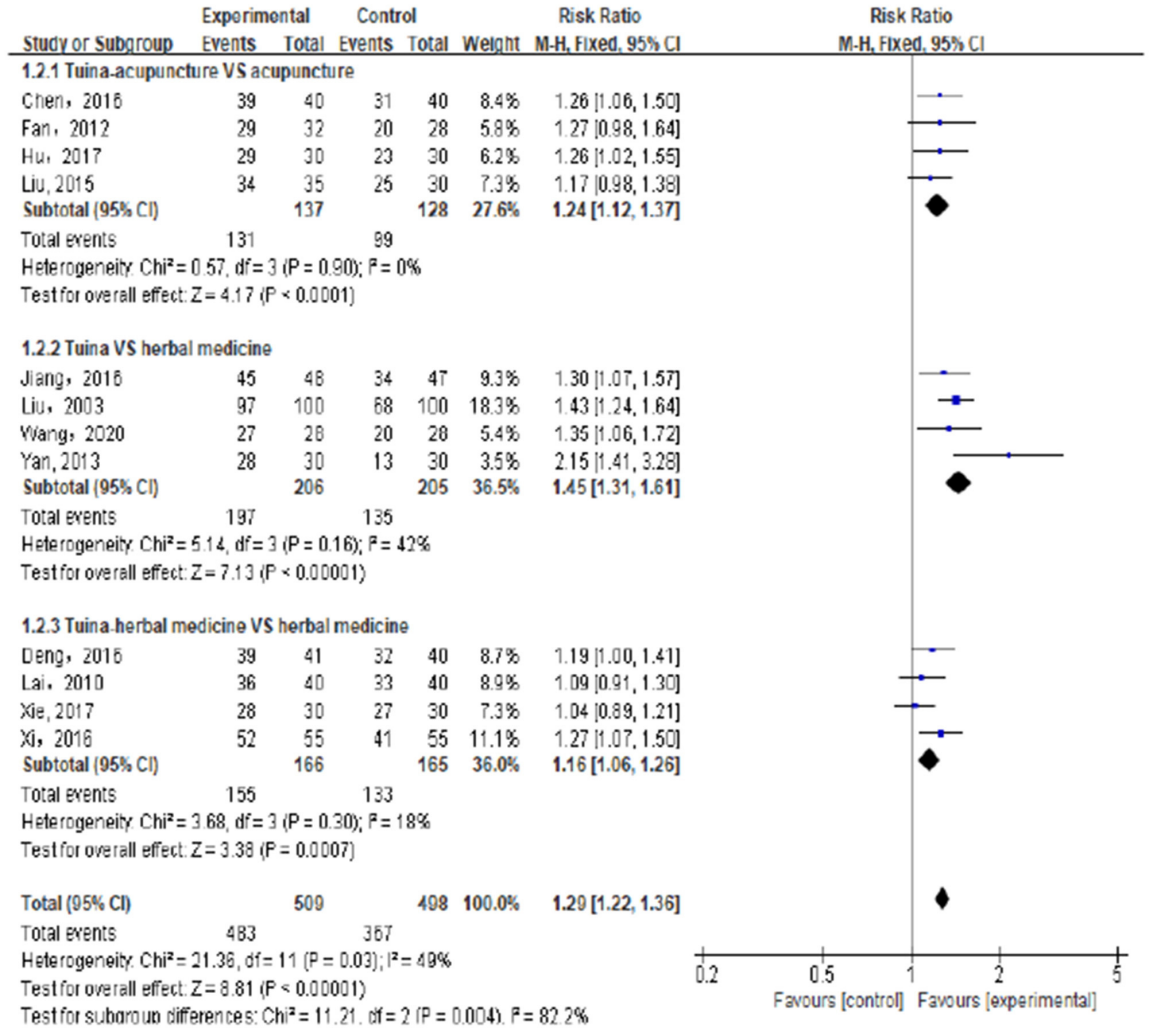
Subgroup analysis showed that compared with the control group, the total effective rate of the experimental group was significantly improved.

Four experiments (21, 23, 24, 37) compared the effective rate of Tuina combined with acupuncture in the treatment of pediatric enuresis. A total of 265 patients were included, including 137 cases in the Tuina plus acupuncture group and 128 cases in the acupuncture group. The results showed that the total effective rate of Tuina plus acupuncture was higher than that of acupuncture alone, and the difference was statistically significant [RR = 1.24, 95%CI (1.12–1.37), *P* < 0.0001]. As shown in Figure 5.

##### Tuina vs. Herbal Medicine

Four experiments (25, 27, 28, 31) compared the effective rates of Tuina vs. herbal medicine in the treatment of enuresis in children. A total of 411 cases were included, 206 cases in the Tuina group and 205 cases in the herbal medicine group. The results showed that the effective rate of the Tuina group was better than that of the herbal medicine group, and the difference was statistically significant [RR = 1.45, 95%CI (1.31–1.61), *P* < 0.00001]. As shown in Figure 5.

##### Tuina Plus Herbal Medicine vs. Herbal Medicine

Four trials (22, 26, 29, 30) compared the effective rates of Tuina combined with herbal medicine vs. herbal medicine alone in the treatment of enuresis in children. A total of 331 cases were included. The results showed that the total effective rate of Tuina combined with herbal medicine was significantly higher than that of herbal medicine alone, and the difference was statistically significant [RR = 1.16, 95%CI (1.06–1.26), *P* = 0.0007]. As shown in Figure 5.

There was no significant heterogeneity for the above subgroup analyses outcomes (*I*^2^ = 0, 42, and 18%, respectively), and the fixed effects model was applied to combine the trials.

### Adverse Reactions

No adverse reactions related to Tuina were reported in the included trials.

### Sensitivity Analysis

Between all included clinical trials, there was generally good homogeneity.

Regarding TER, the primary outcome, the pooled data showed that Tuina, or Tuina combined with other TCM treatments significantly increased TER by 21.2% (RR = 1.29, 95% CI 1.22–1.36, *P* < 0.00001). The results were similar when the sensitivity and subgroup analyses were conducted based on the quality of the study (only included the studies clearly describing randomization methods) (22, 24–26, 29–31) [RR = 1.26, 95%CI (1.17–1.36), *P* < 0.0001], duration of Tuina treatment (≥ 15 days) (22–26, 29, 31) [RR = 1.31, 95%CI (1.20–1.43), *P* < 0.0001]; Year of publication (only studies published in the last 6 years were included) (21, 22, 24, 25, 28–30) [RR = 1.24, 95%CI (1.15-1.33, *P* < 0.00001], subject size (≥ 40 subjects in each group) (21, 22, 25–27, 29) [RR = 1.28, 95%CI (1.20–1.37), *P* < 0.00001]. The results of the sensitivity and subgroup analyses suggested that the results of the meta-analysis were reliable and robust.

### Publication Bias Analysis

**Figure 6:**
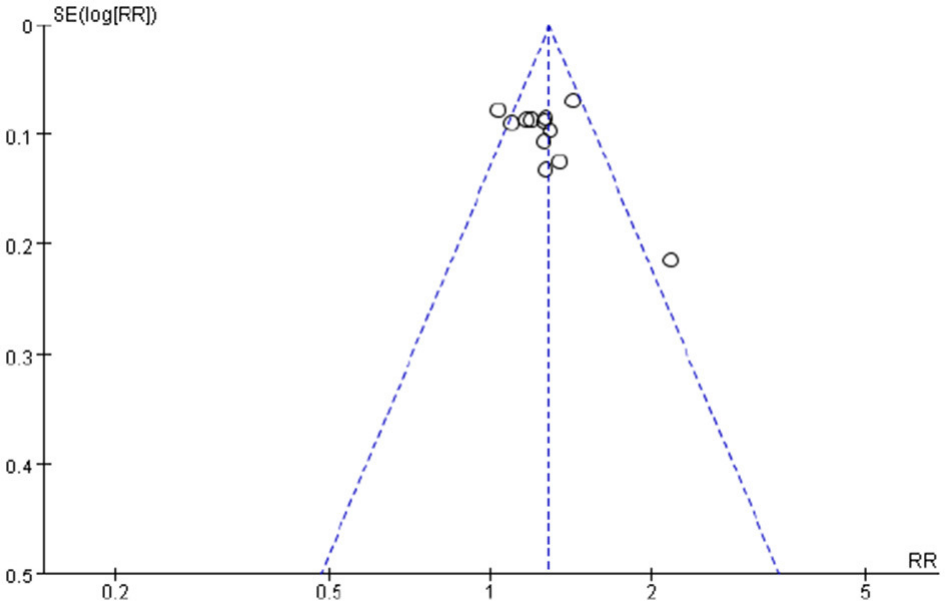
Funnel plots showed publication bias.

The funnel plots of the total effective rate, the primary outcome, suggested possible publication bias in small trials (Figure 6).

## Discussion

According to modern medicine theory, enuresis in children is related to psychological factors, genetic factors, imperfect neurodevelopment (38), abnormal bladder function, and other factors. Invisible spina bifida can also cause bed-wetting in children. In the theory of TCM, enuresis is related to congenital deficiency, the pathogenesis of enuresis in children is complex and its etiology has not yet been fully elucidated. The main etiology and pathogenesis of enuresis in children are the asthenia and coldness of the Xia Yuan (primordial qi of lower energizer), the deficiency of spleen, lung, and kidney, the dampness and heat of the liver meridian, and the incompatibility of the heart and kidney (39). Western medicine treatments mainly include functional exercise, psychological support, and clinical medications (mainly desmopressin) (40, 41). Long-term medications for children may cause greater side effects, such as psychological burdens and even serious psychological disorders. Increasing reports have shown that TCM might play a promising role in the management of children's enuresis, it shows good clinical effectiveness without notable side effects. TCM treatment methods include herbal medicine, acupuncture, Tuina, etc. Tuina has been broadly used for the treatment of enuresis in children, a master's thesis (42) reported a meta-analysis of Tuina in the treatment of enuresis in children (42). The main differences between this thesis and our study included: a) Wen's study included the studies that used other non-TCM treatments, such as western medicine, physical therapy, etc. In our study, only TCM treatments were used for both the control group and the treatment group. b) Wen's study did not evaluate the overall effects of Tuina vs. other treatments, only subgroup analyses were performed, in situations where no overall effects were evaluated, subgroup analyses were unreliable. It was not wise to draw confirmatory conclusions from subgroup findings (43, 44). There is a need to perform a meta-analysis to systematically evaluate the overall effects of Tuina for enuresis in children, and compare the effects of Tuina vs. non-Tuina TCM treatments. The present study is the first systematic review and meta-analysis comparing the difference in the clinical efficacy of Tuina vs. non-Tuina TCM treatments for enuresis in children.

According to the TCM etiology and pathogenesis of enuresis in children, the basic therapeutic principles of Tuina are balancing yin and yang, supplementing the insufficiency and reducing the excess, etc (45). Based on the TCM syndrome differentiation with meridian and acupoints as the outline (46), the individualized treatment regimes are also different, commonly used regimes include Tuina on the acupoints Baihui (GV20) and Qihai (CV6) to warm the yang and dispel cold, pinching the ridge of the Du Channel to facilitate the conduction of qi through the meridians to tonify qi, pushing Tianheshui acupoint (PC9- PC3 direction) to clear the liver and dispel dampness, kneading the Neiguan (PC6) and Shenmen (HT7) acupoints to clear the heart and warm the kidneys, etc. (47, 48).

In this study, 12 RCTs were finally included. The results of this meta-analysis indicate that, compared with non-Tuina TCM treatments, Tuina could significantly improve the total effective rate of enuresis in children by 21.2% [RR = 1.29, 95%CI (1.22–1.36), *P* < 0.00001], no adverse reactions were reported in the included studies, indicating that Tuina appears to be effective and safe for the treatment of enuresis in children and might bring clinical benefits to them.

Enuresis is more common in very young children, so it is also called pediatric enuresis. Although herbal medicine and acupuncture are also effective for pediatric enuresis, herbal medicine is not very suitable for very young kids because it usually tastes bitter, and acupuncture is an invasive treatment. Therefore, as a non-invasive treatment, Tuina is more suitable for young children, Tuina therapy is well tolerated by children and has better compliance for long-term therapy. The results of subgroup analyses showed that Tuina was more effective than herbal medicine in the treatment of enuresis in children (*P*< *0.00001*), Tuina combined with herbal medicine or acupuncture in the treatment of nocturnal enuresis was more effective than herbal medicine (*P* = *0.0007*) or acupuncture (*P*< *0.0001*) alone, suggesting that Tuina is an optimal treatment choice for enuresis in children, if the kids could accept herbal medicine or acupuncture treatment, Tuina combined with herbal medicine, or Tuina combined with acupuncture are also preferred than herbal medicine or acupuncture alone.

This meta-analysis has some limitations:

(a) The quality of 4 included RCTs in this study was not high, and there were potential risks of bias due to the lack of randomization methods, and methods of blinding, etc.
(b) The treatment duration and follow-up time were relatively short, the long-term outcomes of Tuina could not be evaluated.
(c) Individual data were insufficient, subgroup and sensitivity analyses based on country, age, sex, severity, specific method of Tuina, the pathogenesis of enuresis, etc. were not performed.
(d) Adverse reactions were not reported in any included studies. Therefore, only efficacy evaluation can be carried out, and the safety of tuina could not be evaluated. Further studies in this area should be strengthened in the future.
(e) There was potential publication bias.

## Conclusions

From the available evidence, Tuina, or Tuina combined with non-Tuina TCM treatments (acupuncture, or herbal medicine) can improve the clinical outcome of children with enuresis, indicating Tuina is a promising treatment choice for children's enuresis. However, because of the intrinsic limitations of the included studies, more high-quality randomized controlled trials with longer follow-up are still needed to further confirm the efficacy and safety of Tuina in the treatment of nocturnal enuresis in children.

## Data Availability Statement

The original contributions presented in the study are included in the article/Supplementary Material, further inquiries can be directed to the corresponding author/s.

## Author Contributions

CT and QH methodology, investigation, verification, analysis, and manuscript writing. MH verification, investigation, and formal analysis. ZZ writing review and editing. QW and MC verification, investigation, design, review, and supervision. All authors contributed to the article and approved the submitted version.

## Funding

This study was funded by the Science and Technology Development Fund, Macau SAR (file No.: 0010/2019/A, 0099/2018/A3, 0098/2021/A2, and 130/2017/A3), Science and Technology Planning Project of Guangdong Province (2020B1212030008).

## Conflict of Interest

The authors declare that the research was conducted in the absence of any commercial or financial relationships that could be construed as a potential conflict of interest.

## Publisher's Note

All claims expressed in this article are solely those of the authors and do not necessarily represent those of their affiliated organizations, or those of the publisher, the editors and the reviewers. Any product that may be evaluated in this article, or claim that may be made by its manufacturer, is not guaranteed or endorsed by the publisher.
