# Supplementary material for: Tuina for Enuresis in Children: A Systematic Review and Meta-Analysis of Randomized Controlled Trials
Source: Front Public Health. 2022 Apr 12;10:821781. doi: 10.3389/fpubh.2022.821781 (PMC9039245; doi:10.3389/fpubh.2022.821781)
Supplement: Supplementary file 1 [file Data_Sheet_1.ZIP › Supplementary Material/Supplementary_figure 4.docx]

Supplementary Material

# Supplementary Figures 4


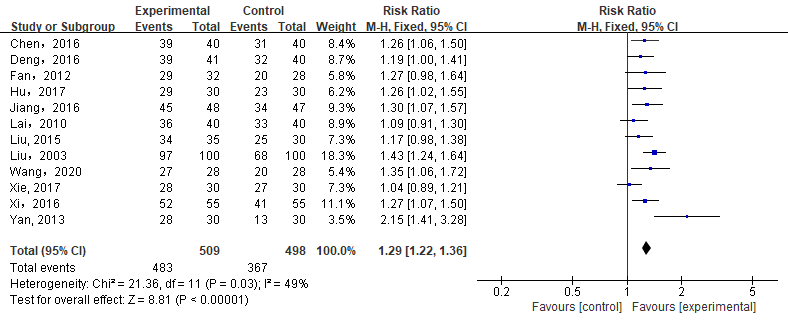


Figure 4. Compared with the control group, the total effective rate of the experimental group was significantly improved.
