# Supplementary material for: Tuina for Enuresis in Children: A Systematic Review and Meta-Analysis of Randomized Controlled Trials
Source: Front Public Health. 2022 Apr 12;10:821781. doi: 10.3389/fpubh.2022.821781 (PMC9039245; doi:10.3389/fpubh.2022.821781)
Supplement: Supplementary file 1 [file Data_Sheet_1.ZIP › Supplementary Material/Supplementary_figure1.docx]

Supplementary Material

# Supplementary Figures

Quantitative synthesis

(Meta-analysis)

(n=12)

Qualitative synthesis

(n=12)

The literature included in the study was reviewed

(n=12)

**Full-text articles excluded (n=48)**

Non-RCT study (n= 28)

Compared with western medicine (n=7)

Case report (n= 6)

Meta analysis (n=1)

Pseudo randomized controlled trials (n=6)

Number of documents obtained after preliminary screening

(n=60)

**Records excluded (n=124)**

Duplicated studies (n=53)

Did not meet the inclusion criteria(n=23)

Clinical experience/observation/ experience (n=36)

Summary and review studies (n=12)

Records identified through database searching

(n=184)

Additional records identified through other sources

(n=0)

**Supplementary Figure 1**. Preferred reporting items for systematic reviews and meta-analysis (PRISMA) search diagram.
