# Supplementary figures and images for: Tuina for Enuresis in Children: A Systematic Review and Meta-Analysis of Randomized Controlled Trials
Source: Front Public Health. 2022 Apr 12;10:821781. doi: 10.3389/fpubh.2022.821781 (PMC9039245; doi:10.3389/fpubh.2022.821781)

Supplementary Material

# Supplementary Figures 2

#
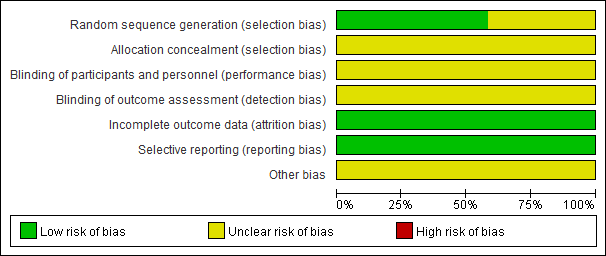


Figure 2. Risk of bias graph

Supplement: Supplementary file 1 [file Data_Sheet_1.ZIP › Supplementary Material/Supplementary_figure 2.docx]

Supplementary Material

# Supplementary Figures 3


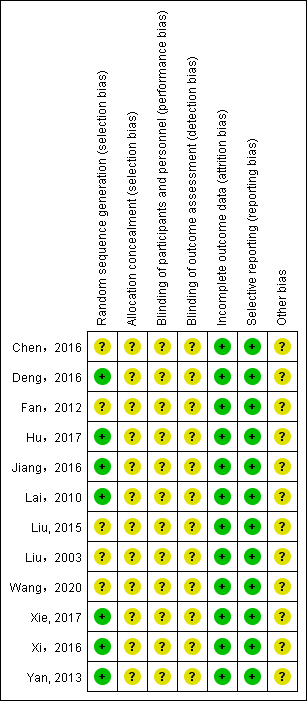


Figure 3. Risk of bias summary

Supplement: Supplementary file 1 [file Data_Sheet_1.ZIP › Supplementary Material/Supplementary_figure 3.docx]

Supplementary Material

# Supplementary Figures 6


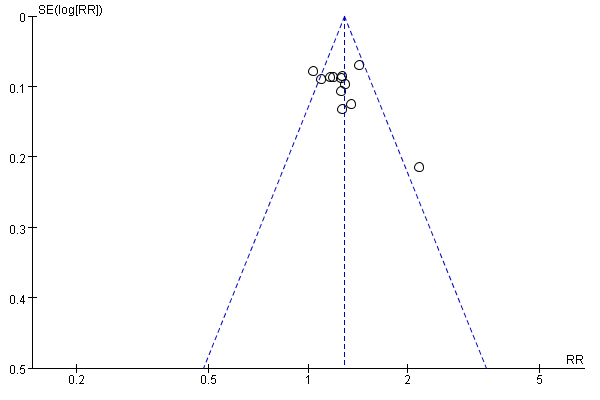


Figure 6. Funnel plots showed publication bias

Supplement: Supplementary file 1 [file Data_Sheet_1.ZIP › Supplementary Material/Supplementary_figure 6.docx]
